# Draft Genome Sequence of Toxigenic *Corynebacterium ulcerans* Strain 04-7514, Isolated from a Dog in France

**DOI:** 10.1128/genomeA.00172-16

**Published:** 2016-03-31

**Authors:** Luis C. Guimarães, Marcus V. C. Viana, Leandro J. Benevides, Diego C. B. Mariano, Adooney A. O. Veras, Pablo H. C. Sá, Flávia S. Rocha, Priscilla C. B. Vilas Boas, Siomar C. Soares, Maria S. Barbosa, Nicole Guiso, Edgar Badell, Adriana R. Carneiro, Vasco Azevedo, Rommel T. J. Ramos, Artur Silva

**Affiliations:** a Institute of Biological Sciences, Federal University of Pará (UFPA), Belém, Pará, Brazil; b Institute of Biological Sciences, Federal University of Minas Gerais (UFMG), Belo Horizonte, Minas Gerais, Brazil; c Department of Immunology, Microbiology and Parasitology, Institute of Biological Sciences and Natural Sciences, Federal University of Triângulo Mineiro (UFTM), Uberaba, Belo Horizonte, Brazil; d Institut Pasteur, Unité de Prévention et Thérapies Moléculaires des Maladies Humaines, National Centre of Reference of Toxigenic Corynebacteria, Paris, France

## Abstract

Here, we present the draft genome of toxigenic *Corynebacterium ulcerans* strain 04-7514. The draft genome has 2,497,845 bp, 2,059 coding sequences, 12 rRNA genes, 46 tRNA genes, 150 pseudogenes, 1 clustered regularly interspaced short palindromic repeat (CRISPR) array, and a G+C content of 53.50%.

## GENOME ANNOUNCEMENT

*Corynebacterium ulcerans* is an important zoonotic bacterium, and case report infections have been increasing worldwide during the last decade (1–3). This bacterium is Gram-positive, nonmotile, pleomorphic, arranged in palisades or V-shaped forms, and non-spore forming. It is facultatively anaerobic, catalase positive, nitrate negative, oxidase negative, and differs from other species of the genus by the fermentation of glycogen and starch (4).

*C. ulcerans* exhibits levels of genomic DNA relatedness with *Corynebacterium diphtheriae* and *Corynebacterium pseudotuberculosis*. Furthermore, taxonomic analyzes of 16S rRNA gene sequences highlight the close phylogenetic relationship between these three species, putting them in a distinct cluster of the genus *Corynebacterium* (1, 4)

Additionally, in 1926, a strain of *C. ulcerans* coding for diphtheria toxin was isolated from the human throat (5). This diphtheria toxin has 95% similarity compared to the diphtheria toxin present in *C. diphtheriae* (2). Nevertheless, nontoxigenic *C. ulcerans* strains have been reported to code for a powerful and severe dermonecrotic toxin similar to phospholipase D from *C. pseudotuberculosis* (6). This repertoire of potent toxins shared for these three species corroborates the apparent relationship between them.

In this study, we present the draft genome sequence of toxigenic *C. ulcerans* strain 04-7514. This strain was isolated from a dog in Bourges, France. The strain is part of the Collection of Institut Pasteur (CIP) (https://www.pasteur.fr/en). These were kindly given to the Laboratory of Genomics and System Biology located at the Federal University of Pará, Belém, Pará, Brazil, and the Laboratory of Cellular and Molecular Genetics located at the Federal University of Minas Gerais, Belo Horizonte, Minas Gerais, Brazil.

The genome sequencing was performed using the next-generation sequencing SOLiD platform, using a fragment library. The predicted genome coverage was approximately 6,000×, based on *C. ulcerans* genomes available in GenBank (http://www.ncbi.nlm.nih.gov/genbank/). The *de novo* assembly strategy was performed using Velvet software version 1.2.10 (7), generating 28 contigs with 2,497,845 bp. The contigs were submitted to GenBank for automatic annotation. The genome has 2,059 coding sequences, 12 rRNA genes, 46 tRNA genes, 150 pseudogenes, 1 clustered regularly interspaced short palindromic repeat (CRISPR) array, and a G+C content of 53.50%. This genome is part of further studies of comparative genomics, pathogenicity, and vaccine and drug targets of the species.

### Nucleotide sequence accession numbers.

The *C. ulcerans* whole-genome shotgun (WGS) project has the project accession no. LJVH00000000. The version described in this paper is version LJVH01000000 and consists of sequences LJVH01000001 to LJVH01000028.
